# Diagnosing growth in low-grade gliomas with and without artificial intelligence-measured longitudinal volume measurements: A retrospective observational study

**DOI:** 10.1093/noajnl/vdaf271

**Published:** 2026-01-06

**Authors:** Hassan M Fathallah-Shaykh, Houman Sotoudeh, Markus Bredel, Alex Whitley, Jinsuh Kim, Fanny E Morón, Fabio Raman, Nidhal Bouaynaya, Hayat Rahal

**Affiliations:** Department of Neurology, The University of Alabama at Birmingham, Birmingham, AL (H.M.F.-S.); Department of Radiology, University of Texas Southwestern, Dallas, TX; Department of Radiation Oncology, University of Miami, Miami, FL; ­Central Alabama Radiation Oncology, Montgomery, AL; Department of Radiology and Imaging Sciences, Emory University, Atlanta, GA; Department of Radiology, Baylor College of Medicine, Houston, TX (F.E.M.); Department of Radiology, Johns Hopkins School of Medicine, Baltimore, MD; Department of Computer and Electrical Engineering, Rowans University, ­Glassboro, NJ; MRIMath, Birmingham, AL

**Keywords:** artificial intelligence, longitudinal imaging, low-grade glioma, volumetric analysis

## Abstract

**Background:**

Low-grade or grade 2 diffuse gliomas (LGG) infiltrate the brains leading to significant neurological morbidity. This retrospective observational study evaluates the ability of AI-assisted volumetric analysis to correctly detect tumor growth in longitudinal studies of LGG as compared to the standard clinical method.

**Methods:**

A total of 56 gliomas and 7 stable FLAIR lesions were included; gliomas were classified as clinical progression (*n* = 34), or clinically stable (*n* = 22). All gliomas were from radiation-naïve patients; only 2 patients had completed treatment with temozolomide. The dates of tumor growth were gathered from clinical notes. Longitudinal tumor volumes were calculated by the MRIMath FLAIR AI. Golden truths were obtained by physician reviews using the MRIMath Smart contouring system. Growth by significant shifts in tumor volumes was detected by using the statistical method of online change-of-point method.

**Results:**

In the clinical progression group, automatic AI segmentation followed by human review detected tumor growth at a median of 21 months earlier than visual inspection. In the clinically stable group, AI with human review identified growth in 13/22 cases at a median of 23 months earlier than the last magnetic resonance imaging. AI without human review generated similar results but with a 25% false positive and an 8.33% false negative rate. The median time spent by physicians in reviewing, revising, and approving the AI segmentations is 2 minutes.

**Conclusions:**

These findings highlight the clinical potential of AI-assisted volumetric analysis followed by physician oversight for the timely detection of tumor progression in LGG patients.

Key Points

- AI-supported, physician-reviewed volumetric analysis of low-grade gliomas enables earlier detection of tumor growth compared with standard clinical practice.
- A National Cancer Institute-funded, 510(k)-cleared AI-supported online device integrates efficiently into routine neuro-oncology workflows.
- AI-generated contours and volumetric measurements are produced within seconds, with physician review completed in 2-3 minutes.

Importance of the Study

Low-grade (WHO grade 2) diffuse gliomas (LGG) infiltrate the brains of young, otherwise productive adults and cause substantial neurological morbidity. Because volumetric tumor measurements have historically been time-consuming and labor-intensive, current clinical practice largely relies on qualitative visual assessment by doctors. To address this, the National Cancer Institute supported the development of an FDA-cleared AI-enabled online platform that rapidly measures tumor volume and provides physician-reviewed results within minutes. In our study, combining AI with physician oversight allowed tumor growth to be detected much earlier—on average nearly 2 years sooner—than with standard visual assessment alone. In several cases, the AI-assisted approach identified progression that had been previously considered stable. While the AI alone performs better than standard care, the most accurate results are achieved when physicians review the AI output. This technology could help doctors recognize tumor changes sooner and make more informed decisions about patient care.

Low-grade gliomas (LGGs) represent a significant challenge due to their subtle growth patterns and the difficulty of early and accurate detection of tumor progression. LGG account for approximately 15% of all gliomas, with an incidence rate of about 1 in 100,000. These tumors are most observed in adults in their 30s and 40s.^1^ LGGs, classified as WHO grade 2 gliomas, constitute a considerable proportion of adult brain tumors and are known for their brain invasion and potential for malignant transformation; the median life expectancy of an IDHmt astrocytoma grade 2 is between 10-15 years, and for an oligodendroglioma slightly longer.^1^ The predominant majority of LGG carry mutations in the IDH gene, which would make them potentially susceptible to clinically proven IDH inhibitors.^2^ The incidence of LGGs transforming into higher-grade tumors has been reported to be as high as 72%.^3^ The prognosis of grade 2 IDH-mutant astrocytomas is only slightly better than grade 3, with median survival of 11 years compared to 9 years.^3^^,^^4^

The current standard of care for treating LGG includes a multidisciplinary approach tailored to individual patient factors, such as tumor location, genetic markers, and patient age. Surgical resection is typically the initial treatment, with the goal of maximal safe removal of the tumor to alleviate symptoms and obtain tissue for histopathological and molecular analysis.^5^ Postoperative management protocols are guided by molecular markers such as IDH mutation and 1p/19q codeletion status, which have prognostic significance. For high-risk patients (eg, subtotal resection, or unfavorable molecular profile) adjuvant therapies are considered. Post-radiation chemotherapy is often recommended to improve progression-free and overall survival.^6^ Regardless of the treatment protocol and molecular profile, the clinical management of LGGs typically relies on regular monitoring by longitudinal magnetic resonance imaging (MRI) to detect progression. Traditionally, visual inspection by radiologists has been the clinical gold standard for assessing tumor growth over time. However, this method is inherently subjective and prone to inter-observer variability, often resulting in delayed detection of growth, which can impact patient outcomes.^7^

Traditionally, longitudinal studies of LGGs have been evaluated using visual inspection or 2D diameter measurements, which have been the standard technique for assessment according to the Response Assessment in Neuro-Oncology (RANO) criteria.^8^ So far, volumetric measurements have not been widely used because manual contouring methods are time-consuming and prone to high inter-user variability. Recent advances in artificial intelligence bring timely and accurate volumetric measurements within reach.^7^^,^^9^ There has been a gradual, though slow, shift from 2D measurements to 3D/volumetric assessments of LGGs. Additionally, the most recent version of the RANO criteria, RANO 2.0 (introduced in 2023), now incorporates 2D, 3D, and volumetric measurements. It also allows for AI-based segmentation, with human supervision during the process.^10^

Here, we evaluate the efficacy of volumetric analysis using the MRIMath platform, including a FLAIR AI and human supervision using the Smart contouring system, in detecting tumor growth in LGG patients as compared to standard visual inspection. We study AI alone and AI combined with human review and approval. Previous studies have shown the benefits of volumetric analysis combined with the online change-of-point statistical method in detecting tumor growth significantly earlier than visual inspection.^11^ Fathallah-Shaykh et al. applied the computationally intensive method of non-negative matrix factorization to segment LGG^12^; here we use an accurate and efficient AI that generates segmentations in seconds. We also evaluate the role of physician review in enhancing AI prediction accuracy. We hypothesize that AI with volumetric assessment allows for earlier detection of tumor growth than current clinical standard of radiological assessment. For volumetric assessment, we apply the statistical change-of-point method to determine the first point of growth. The change-of-point method applies the same rigorous statistical standard to all patients and studies and determines if a current measurement is significantly different from all the measurements of the same patient.^7^^, ^^9^

## Methods

### Ethical Approval

The Institutional Review Board of the University of Alabama at Birmingham approved the research (IRB-150618007); waiver of informed consent was granted because the research involved no greater than minimal risk and no procedures for which written consent is normally required outside the research context.

### Study Design and Patient Selection

The patients were seen at the neuro-oncology clinics of the University of Alabama at Birmingham between July 1, 2017, and May 14, 2018. The inclusion criteria were (1) pathological diagnosis of grade 2 oligodendroglioma (oligo), grade 2 astrocytoma (astro), or grade 2 mixed glioma (oligostarocytoma) in the brain excluding the pineal gland and (2) either no postoperative treatments or chemotherapy with temozolomide, (3) at least 4 MRI scans available for review either after the initial diagnosis or after the completion of chemotherapy with temozolomide (if applicable). We include mixed gliomas because of the retrospective nature of this study. The exclusion criteria were (1) treatment with radiation therapy after the initial diagnosis or (2) radiological reports indicating development of new enhancement without an increase in FLAIR signal. Patients treated by radiation therapy were excluded because radiation may confound the results by causing an independent increase in FLAIR signal. We excluded patients whose radiological reports described new enhancing nodules without an increase in FLAIR signal because they are easily detected by visual examination.

A total of 56 gliomas met the inclusion criteria, including 22 oligodendrogliomas, 30 astrocytomas, and 4 mixed gliomas; only 2 patients received temozolomide. All of the oligos had the 1p/19q co-deletions except for 1 with a single deletion of 19q. The pathological reports for the 4 gliomas classified as mixed lacked molecular data necessary for reclassification. At the time of retrospective review, 34/56 patients had been diagnosed with clinical progression (ie, clinical progression group) while the remaining 22/56 were diagnosed as being clinically stable (clinically stable group) by visual comparison of the most recent MRI performed at the last clinic visit. We reviewed the records of 8 patients monitored for imaging abnormalities without a pathological diagnosis. One patient was excluded due to insufficient follow-up data. After appropriate follow-up with a mean of 6 years, clinicians determined that the imaging abnormalities in the remaining 7 patients were unlikely to represent gliomas. Their ages range from 45 to 68 with a mean of 59 years. To ensure methodological rigor, these 7 cases were included as negative controls in the application of the change-point method to measured volumes. As no surgical intervention was performed, a pathological diagnosis could not be established. The most likely diagnosis was demyelination; these lesions were not attributable to vascular malformations or postoperative changes. The same AI pipeline was applied to the 7 patients in the stable FLAIR (negative control) group.

The selection criteria required at least 4 MRI scans, excluding those who received radiation therapy post-diagnosis, to ensure consistency in the study population. The final dataset included 63 patients divided into 3 groups: clinical progression (*n* = 34), clinically stable (*n* = 22), and imaging abnormality (*n* = 7).

### Time to Growth Detected by Standard Clinical Care

Both the radiologist and neuro-oncologist had access to the pathological findings through the medical records. Radiological reports were generated by various board-certified neuroradiologists at the University of Alabama at Birmingham Hospital after reviewing each longitudinal MRI scan. These reports did not specify whether the current scans were compared with postoperative MRIs and the radiologists did not include any 2D measurements in their reports. Neuro-oncology notes were also reviewed retrospectively. Clinical progression was defined by a radiological report indicating tumor growth in conjunction with a neuro-oncology note documenting communication of this change to the patient or family. The time to growth detection was retrospectively calculated based on the impressions recorded in the radiological reports. Because of the retrospective nature of this study, the radiologists and neuro-oncologists were blinded to the AI outputs.

### The MRIMath Cyber Device

The MRIMath i^2^Contour cyber device has received 510k approval from the Food and Drug administration (www.mrimath.com).^13^^,^^14^ The platform establishes a secure connection with a picture archiving and communication system or folder located behind institutional firewall for automatic transfer of a study in seconds. Users may: (1) login by 2-factor authentication or single sign-on from any location using a chrome browser, (2) upload data, (3) view dicom files, (4) order AI segmentation for GBM T1c or FLAIR sequences and obtain results in seconds, (5) review and make changes using the MRIMath low-variability Smart contouring system,^9^ (6) view volume plots of longitudinal studies, and (7) download both volumetric data and segmentations.

MRI scans were uploaded to the MRIMath platform and segmented by the GBM FLAIR AI, trained on both pre- and post-operative segmented FLAIR images; the mean overall Dice-Sørensen coefficient (DICE) score of the AI as compared to a common ground truth derived from 3 independent neuro-radiologists was 92%^9^; a description of the training of the MRIMath AI and how it compares to other segmentation systems is detailed at.^9^ We have used Version 1.0. The input of the AI is one slice at a time. While the MRIMath AI models are primarily trained on 2D slices, they include post-processing steps that incorporate 3D spatial continuity and contextual information. The parameters of the trained AI model are fixed.

The MRIMath platform includes a Smart Contouring system that enables physicians to review, revise, and approve the AI-generated segmentations. MRIMath© adopts a streamlined approach by handling a single series type (FLAIR or T1c), which contrasts with the modality integration and subcomponent segmentations used by the other platforms.^15^^,^^16^

### Tumor Segmentation, Physician Review, and Volume Calculations

A total of 653 MRIs were uploaded to the MRIMath platform (the dicom headers are summarized in Supplementary ­Information). Segmentation results were reviewed by 98 physicians, including neuroradiologists (*n* = 21), neuro-oncologists (*n* = 14), radiation oncologists (*n* = 60), and neurosurgeons (*n* = 3). The reviewers included 31 physicians in private practice, 16 residents, 5 fellows, 24 assistant professors, 13 associate professors, and 9 professors. Physician reviews served as the gold standard for calculating the average dice score for evaluating the accuracy of the AI-generated manual segmentations. Tumor volumes were calculated from the segmented tumors. Inter-user variability was measured from the cases with more than one reviewer by calculating the average Dice score by taking one reviewer as the gold standard. Intra-user variability was measured by computing the average Dice score between 13 MRIs approved by 2 physicians 18 months apart.

### Online Change-of-Point Detection

To detect the first significant change in a series of longitudinal volumes, we apply the online change-of-point method to the AI-generated measurements with or without human review. To exclude FLAIR changes due to the evolution of post-surgical changes, the baseline volume in the longitudinal series was the first minimum after surgical resection. To identify an abrupt change of volume, we applied a change in the root-mean-square level at a minimum threshold of 500/(volume at baseline) and a minimum of 2 samples between change points. The number 500 corresponds to 5% of the rounded median of the baseline volume. The change-point MATLAB (Natick, MA) function is provided in the Supplementary Material.

The change-of-point is a statistical technique used to identify points in a time series where the data’s properties, such as mean, variance, or distribution, change significantly.^17^^,^^18^ These points, known as change points, indicate shifts in the underlying process generating the data. Detecting such changes is crucial in patient follow up and can help the physician to detect subtle changes of MR images. There are 2 primary approaches to change point detection: the online method detects changes in points in real-time as new data becomes available, making it essential for applications requiring immediate detection and response. The offline method analyzes a complete dataset to identify any change points that have occurred. It’s often used in post hoc analyses where the entire data sequence is available.^19^ Here, we use the algorithms developed by Rebecca Killick and apply the online method, as it replicates clinical scenarios where the volumetric series includes only measurements taken before a specific date.^20^

## Results

### Review Time, Dice Scores, and Variability

The median time spent reviewing, revising and approving the AI segmentations was 1.18 minutes (lower quartile of 1.18 minutes and an upper quartile of 3.31), mean = 2.93 minutes, 95% CI: (2.32, 3.54). The average DICE score of the FLAIR AI predictions, as compared to the golden truths segmentations approved by physicians, is 87.7. Fifty-one cases were reviewed by more than one physician; the average inter-user DICE score is 87.5, corresponding to a variability of 12.5. The average intra-user DICE score is 93.1, which corresponds to a variability of 6.9%.

### Clinical Progression Group

**Figure 1.**
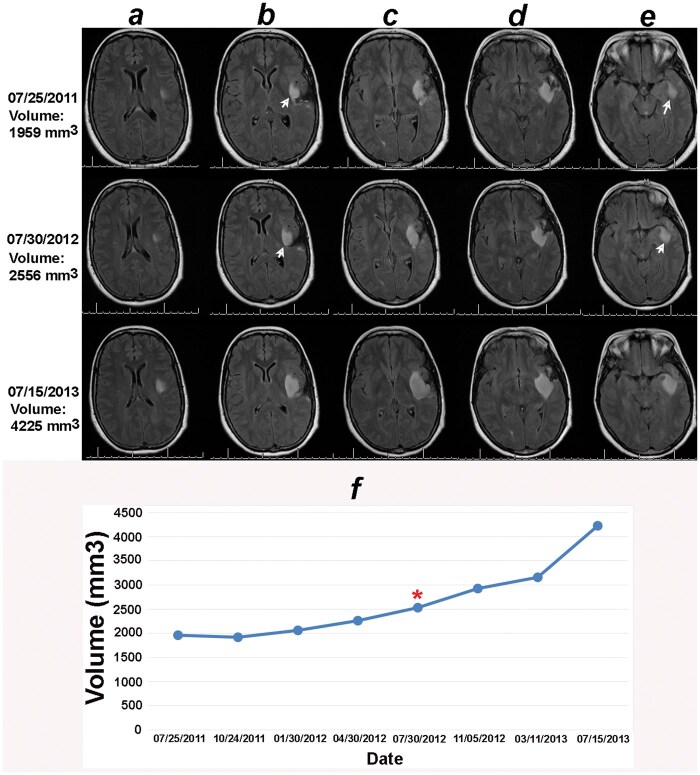
Volumetric analysis combined with the online change-of-point statistical method detects progression on 30 July 2012, 1 year earlier than the date of progression documented in the clinical notes (15 July 2013). Columns A-E correspond to sections within the same MRI series. Notice that though the tumor in the 2D sections containing the largest tumors under columns (C) and (D) did not change between the baseline (25 July 2011) and 30 July 2012, the 2D sections in columns B and E increased in size between the baseline MRI of 07/25/2011 and the date of progression detected by the change-of-point method, 30 July 3012 (white arrows). (F) The online change-of-point analysis detects progression at the red Asterix. The clinical notes detected progression 1 year later.

The clinical progression group includes the LGG patients whose last MRIs were interpreted as showing progression by the radiological reports. The results demonstrate that volumetric analysis calculated by AI segmentation followed by human review detects tumor growth significantly earlier than traditional visual inspection by radiologists (median of 21 months, Table 1, and Figure 1). Conversely, AI without human review also detects tumor growth earlier than visual inspection (median of 21 months), but with false negative and positive results (Table 1); it missed 3 out of 35 cases, and these misses were primarily associated with smaller tumors that were more challenging to detect and segment.

**Table 1.** Detecting tumor growth in the Clinical Progression Group earlier than visual inspection by radiologists

Measure	AI without human review	AI with human review
Median (months)	21	21
Lower interquartile range (IQR)	11.75	9
Upper IQR	38.75	31.5

### Clinically Stable Group

**Figure 2.**
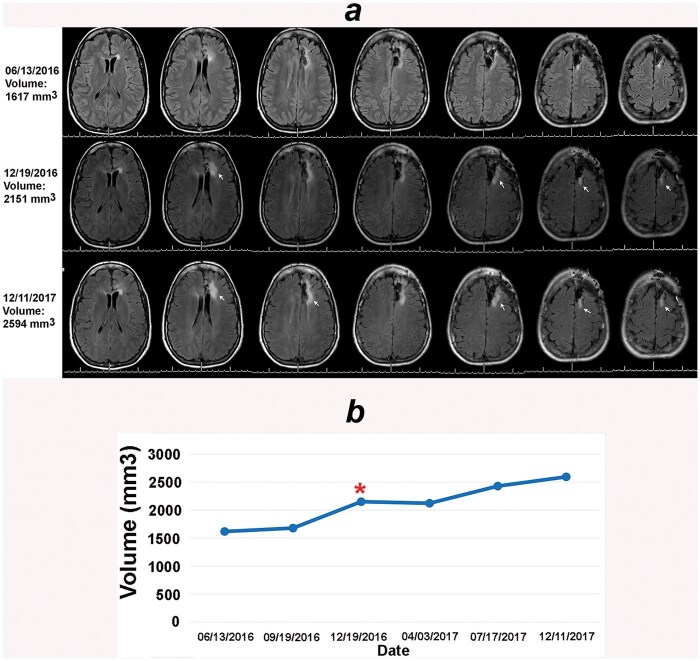
Volumetric analysis combined with the online change-of-point method detected progression on 19 December 2016 (see arrows) as compared to baseline on 13 June 2016. The columns in (A) represent sequential sections from the same MRI series. The MRI on 11 December 2017 was read as stable; arrows point to tumor growth as compared to the MRI of 19 December 2016. (B) The online change-of-point analysis detects progression at the red Asterix. The clinical notes considered all these MRIs as stable.

The clinically stable group includes patients whose last MRI was deemed stable by visual inspection. Volumetric analysis aided by the AI and reviewed by a human, detected tumor growth in 13 out of 22 cases (Table 2 and Figure 2). Remarkably, this detection occurred at a median of 23 months earlier than the most recent MRI scan. In comparison, AI without human review detected growth at a median of 26 months earlier but missed 1 out of the 13 cases. Moreover, while AI with human review accurately flagged the remaining 9 cases as stable, AI without review incorrectly detected tumor growth in 1 of those 9 cases.

**Table 2.** Detecting growth in the Clinically-Stable Group earlier than the last MRI

Measure	AI without human review	AI with human review
Median (months)	26	23
Lower IQR	11.75	9
Upper IQR	67.5	67

### Negative Control Group

The negative control group consisted of FLAIR lesions monitored without a pathological cancer diagnosis. The performance of the volumetric analysis using the change-point method varied significantly depending on whether human review was involved. When used in conjunction with physician review, the system correctly identified all 7 cases as stable, demonstrating perfect specificity. In contrast, without human oversight, 3 out of 7 cases were incorrectly flagged as tumor growth.

### False Negative and False Positive Rates

#### False negative rates

By combining the 35 cases from the clinical progression group and the 13 cases from the clinically stable group that showed progression after computer-assisted diagnosis, the sensitivity the AI without human review missed 4 of these 48 cases (8.33%, Table 3). The variability in the volume measurements caused the change-point method to identify statistically significant changes at different time points.

**Table 3.** False positive and negative rates (per patient)

Method	FP rate	FN rate
AI without human review	25% (4/16)	8.33% (4/48)

#### False positive rates

By combining the 7 cases from the negative control group and 9 stable cases from the clinically stable group, we find that the AI without human review incorrectly flagged a total of 4/16 cases as tumor growth (Table 3).

## Discussion

We find that human-supervised, AI-supported measurements of longitudinal LGG volumes detect tumor growth significantly earlier than radiologists’ interpretations based solely on visual inspection. Furthermore, AI-generated segmentations—without human review—also identify progression dates earlier than visual inspection, although this comes at the cost of false positives and negatives, underscoring the importance of physician oversight.

We segment the LGG using the MRIMath© FLAIR AI, which is FDA-approved for glioblastoma^13^^,^^14^; the “golden truth” is established by Board-certified physicians who review the AI-generated segmentations using the FDA-approved MRIMath© Smart Contouring platform. Our design, involving multiple reviewers from different subspecialties, mirrors real-world clinical scenarios. For volumetric assessment, we employed the statistical online change-of-point method to determine the first point of growth.^7^ The change-of-point method applies a consistent and rigorous statistical standard across all patients and studies. Unlike the conventional RANO product rule, which uses a universal approach based on the product of diameters, the change-of-point method assesses whether a current measurement significantly deviates from all previous measurements for the same patient. The variability between the AI-generated predictions and physician-approved contours (DICE score: 87.7) is comparable to the variability observed between different users (DICE score: 87.5). Additionally, the average intra-user DICE score is 93.1. Notably, when the same AI model is applied to glioblastoma FLAIR images, it achieves a similar DICE score of 92.^9^ Together, these findings highlight both the inherent ­difficulty of precisely delineating FLAIR lesion boundaries and the high accuracy of the AI system in segmenting LGG. In addition, this AI framework can be utilized to track FLAIR changes in post-radiation patients by identifying and quantifying the typical range of treatment-related volumetric alterations.

Most clinical radiologists use visual inspection to evaluate longitudinal studies, while the RANO criteria remain the current standard for detecting progression in clinical trial settings.^21^ Significant limitations of the RANO criteria include large inter-user variability in determining the perpendicular diameters of the largest tumor cross-section and the reliance on 2-dimensional measurements which may not accurately represent the complex 3-dimensional tumor architecture.^11^^,^^22^ Volumetric analysis was also shown to be superior to other established tumor classification criteria, such as the Response Evaluation Criteria in Solid Tumors (RECIST) and modified RECIST,^23^ due to their tendency to oversimplify a multidimensional and heterogeneous tumor.^24^

The goal of this study is to measure and analyze LGG volumes longitudinally. FLAIR volumes are typically larger than T2 and there is a mismatch between FLAIR and T2 volumes in LGG.^25^^,^^26^ We have chosen FLAIR because we believe it reflects that most accurate volume of brain infiltration. None of the tumors showed enhancement on T1c. Volumetric measurement remains the gold standard for assessing the growth of LGGs and detecting progression. In particular, tumor volumes and growth rates serve as key prognostic factors.^27^ One limitation of the present study is the lack of a parallel neuroradiologist-led review of serial MRI examinations according to RANO criteria for determining disease progression. Several studies have reported improved diagnostic accuracy of volumetric analysis as compared to the RANO criteria and radiologists’ interpretations of longitudinal LGG imaging. Fabio et al.report that volumetric measurements detect progression at significantly earlier time points than the RANO criteria^7^^,^^11^; as compared to volumetric ground truth, the accuracy of RANO compared to previous and baseline scans was 21.0% and 56.5%, with an AUC of 0.39 and 0.55, respectively. A recent analysis of the Phase I trial of ivosidenib for LGG also confirmed that 3D volumetric measurements outperform 2D measurements in response assessment with higher inter-reader agreement and lower rates of reader discordance.^28^ The findings of 2 studies on pediatric gliomas support the conclusion that volumetric analysis is more effective than 2D measurements in diagnosing tumor progression at significantly earlier time points.^29^^,^^30^ In a study of 6 patients with pediatric glioma, Khalili et al. reported that volumetric analysis detected progression earlier than radiologists’ interpretations.^30^ Similarly, in a study of recurrent gliomas, Dempsey et al. found that only volumetric measurements of tumor size were predictive of survival, unlike 1D, 2D, and 3D measurements.^31^ One study compared one-dimensional (1D), two-dimensional (2D), and three-dimensional (3D) linear measurements with manual volume measurements in the follow-up of LGG. The authors concluded that 3D “linear” measurement of LGGs is superior to 1D and 2D methods, aligning well with tumor volume calculations, although it is not without limitations.^32^

Several methods are available for automatically assessing the volumes of gliomas. Compared to these methods, the MRIMath FLAIR AI stands out for being fully automated, as it does not require preprocessing steps that involve human supervision, such as deboning, interpolation, or registration. Additionally, while MRIMath© processes images in 2D and treats the FLAIR modality independently, other methods use a 3D approach and integrate the 4 modalities: T1, T1c, T2, and FLAIR.^18^^,^^33^ The MRIMath© FLAIR series AI differs from the subcomponent segmentations employed by other platforms. This study focuses on glioma patients who have not received radiation therapy; nonetheless, our methodology is also valuable in patients who had received radiation, in whom white matter changes on FLAIR could be treatment related.

Limitations of our study include a retrospective design, a small dataset from a single institution, using multiple reviewing physicians from different specialties, primarily using FLAIR sequences, and a comparison to visual inspection. Our goal is to evaluate the importance of AI volumetric analysis in real world scenarios where multiple physicians evaluate longitudinal LGG images.

## Conclusions

Our study highlights the effectiveness of AI-assisted volumetric analysis using the MRIMath FLAIR AI platform for detecting tumor growth in LGGs. By integrating AI with physician review, we were able to detect tumor progression at significantly earlier time points compared to traditional visual inspection methods. This study underscores the potential of AI in clinical oncology, particularly in enhancing the early detection of tumor growth, while also emphasizing the importance of human review in conjunction with a low-variability platform.

## Supplementary Material

vdaf271_Supplementary_Data

## Data Availability

The data that support the findings of this study are available from the corresponding authors upon reasonable request. Restrictions apply to the clinical imaging data used in this study to protect patient privacy, and therefore these data cannot be publicly shared.

## Lay Summary

Low-grade (WHO grade 2) diffuse gliomas infiltrate the brains of young, otherwise productive adults and cause substantial neurological morbidity. Because volumetric tumor measurements have historically been time-consuming and labor-intensive, current clinical practice largely relies on qualitative visual assessment by doctors. To address this, the National Cancer Institute supported the development of an FDA-cleared AI-enabled online platform that rapidly measures tumor volume and provides physician-reviewed results within minutes. In our study, combining AI with physician oversight allowed tumor growth to be detected much earlier—on average nearly two years sooner—than with standard visual assessment alone. In several cases, the AI-assisted approach identified progression that had been previously considered stable. While the AI alone performs better than standard care, the most accurate results are achieved when physicians review the AI output. This technology could help doctors recognize tumor changes sooner and make more informed decisions about patient care.
