# Supplementary material for: Diagnosing growth in low-grade gliomas with and without artificial intelligence-measured longitudinal volume measurements: A retrospective observational study
Source: Neurooncol Adv. 2026 Jan 6;8(1):vdaf271. doi: 10.1093/noajnl/vdaf271 (PMC12909261; doi:10.1093/noajnl/vdaf271)
Supplement: vdaf271_Supplementary_Data [file vdaf271_supplementary_data.docx]

SUPPLEMENTARY INFORMATION

# **Diagnosing growth in low-grade gliomas with and without AI-measured longitudinal volume measurements: A retrospective observational study**

Hassan M Fathallah-Shaykh ^1,^*, Houman Sotoudeh ^2^*, Markus Bredel ^3^, Alex Whitley ^4^, Jinsuh Kim ^5^, Fanny E. Morón ^6^, Fabio Raman ^7^, Nidhal Bouaynaya^8^, and Hayat Rahal^9^

|  |
| --- |

^1^ The University of Alabama at Birmingham, Birmingham, AL; hfshaykh@uabmc.edu

^2^ University of Texas Southwestern, Department of Radiology, Dallas, TX; houman.sotoudeh@utsouthwestern.edu

^3^ University of Miami, Department of Radiation Oncology,Miami, FL; mbredel@miami.edu

^4^ Central Alabama Radiation Oncology, Montgomery, AL; whitley.alex@gmail.com

^5^ Emory University, Department of Radiology and Imaging Sciences, Atlanta, GA; jinsuh.kim@emory.edu

^6^ Baylor College of Medicine, Department of Neurology, Houston, TX; fmoron@bcm.edu

^7^ Johns Hopkins School of Medicine, Department of Radiology, Baltimore, MD; framan1@jh.edu

^8^ Rowans University, Department of Computer and Electrical Engineering, Glassboro, NJ; bouaynaya@rowan.edu

^9^ MRIMath, Birmingham, AL; hrahal@mrimath.com

***** Correspondence: hfshaykh@uabmc.edu; Tel. 205-934-1813; (HFS), [houman.sotoudeh@utsouthwestern.edu](mailto:houman.sotoudeh@utsouthwestern.edu) (HS)


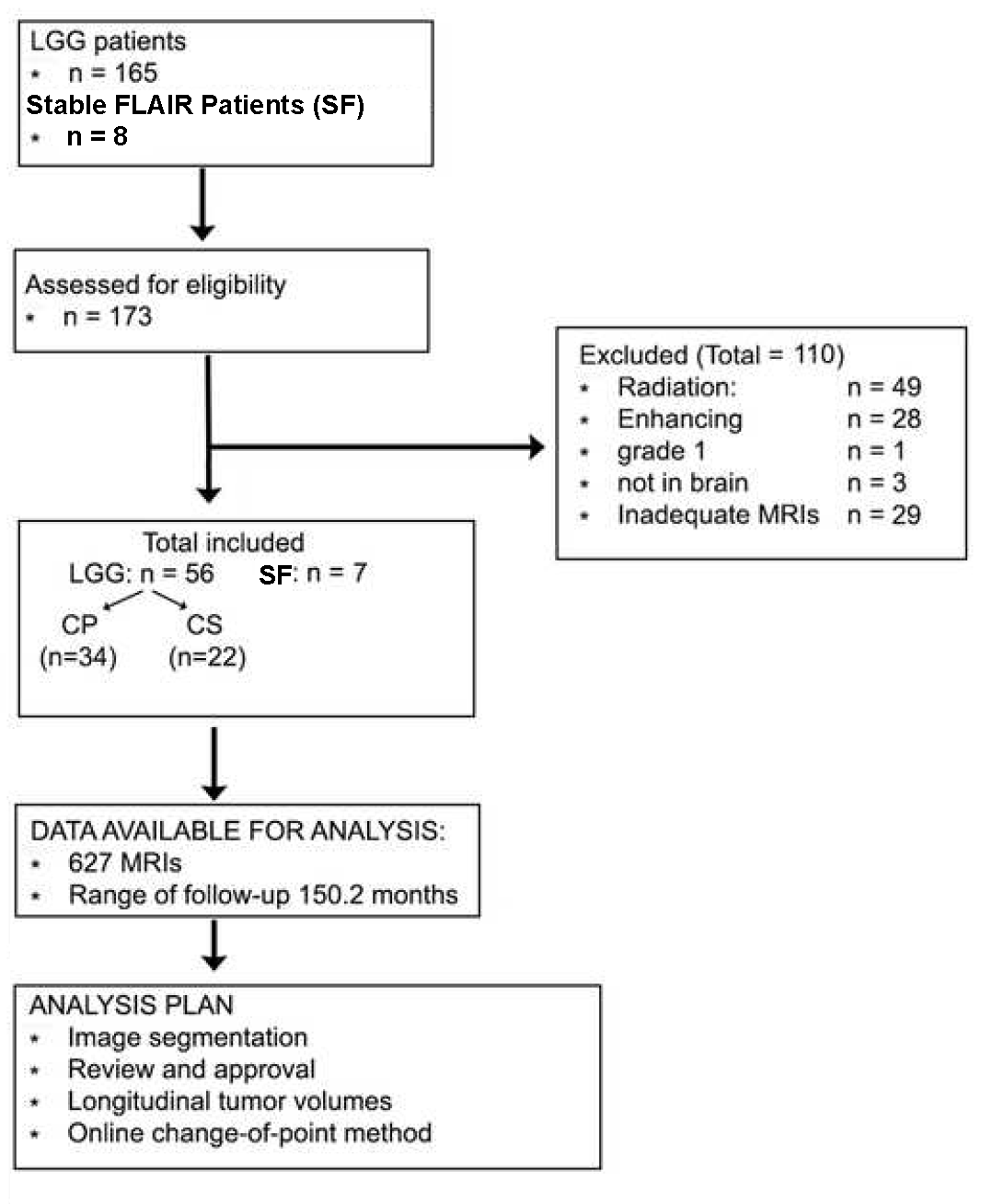


Flow diagram illustrating patient screening and selection according to inclusion and exclusion criteria.

**Online Change of Point Method**

The change of point method was applied using the following matlab function:

findchangepts(x,'Statistic','rms','MinThreshold', min_thresh,'MinDistance', 2). where min_thresh = min(0.1, medbase ./ x(1))

## FLAIR Series Imaging Characteristics

- *Flair Series MRI Manufacturer Distribution*

- GE MEDICAL SYSTEMS: **340**  occurrences

- Philips Medical Systems: **285** occurrences

- SIEMENS: **18** occurrences

- Philips Healthcare: **7**  occurrences

- HITACHI MEDICAL CORPORATION: **3** occurrences

- *Flair Series MRI Manufacturer's Model Name Distribution*

- Achieva: **228** occurrences

- C!: **1** occurrence

- Espree: **4** occurrences

- GENESIS_SIGNA: **40** occurrences

- Ingenia: **49** occurrences

- Intera: **1** occurrence

- Intera Achieva: **14** occurrences

- MAGNETOM EXPERT: **6** occurrences

- Optima MR450w: **2** occurrences

- Prisma: **2** occurrences

- SIGNA EXCITE: **52** occurrences

- SIGNA HDx: **10** occurrences

- Signa HDxt: **229** occurrences

- Symphony: **3** occurrences

- Verio: **2** occurrences

- Unspecified: **10** occurrences

- *Flair series MRI Magnetic Field Strength (Tesla) Used*

- 3.0: **160** occurrences

- 1.5: **482** occurrences

- 0.3: **3** occurrences

- 0.3425: **1** occurrence

- 0.95: **6** occurrences

- 0.7: **1** occurrence

- *Flair Series MR Acquisition Type Distribution*

- 3D: **8** occurrences

- 2D: **583** occurrences

- Unspecified: **62** occurrences

**Table 1:** Flair Series MR Imaging Parameters Statistics

| **Column** | **Average** | **Standard Deviation** | **Median** | **Interquartile Range (IQR)** | **Minimum** | **Maximum** |
| --- | --- | --- | --- | --- | --- | --- |
| **Slice Thickness (mm)** | 4.93 | 0.42 | 5 | 0 | 1 | 6 |
| **Repetition Time (ms)** | 9,698.19 | 1,262.22 | 9,002 | 2,198 | 3,900 | 12,000 |
| **Echo Time (ms)** | 138.74 | 20.72 | 140 | 21.02 | 81.00 | 325.45 |
| **Spacing Between Slices (mm)** | 6.36 | 0.73 | 6.5 | 0 | 1.80 | 7.5 |
| **Inversion Time (ms)** | 2,455.82 | 317.44 | 2,200 | 600 | 1400 | 2,847.39 |
| **Pixel Bandwidth** | 229.79 | 110.96 | 162.73 | 144.27 | 80.00 | 1,246.00 |
| **Echo Train Length** | 21.03 | 25.49 | 1 | 30 | 0 | 182 |
| **Imaging Frequency (MHz)** | 78.95 | 28.20 | 63.88 | 0.04 | 12.71 | 128.17 |
| **Flip Angle (Degrees)** | 92.08 | 13.22 | 90 | 0 | 0 | 180 |
| **Number of slices** | 25.28 | 14.45 | 24 | 2 | 6 | 346 |
